# Supplementary material for: Genotype Characterization and MiRNA Expression Profiling in Usher Syndrome Cell Lines
Source: Int J Mol Sci. 2024 Sep 17;25(18):9993. doi: 10.3390/ijms25189993 (PMC11432263; doi:10.3390/ijms25189993)
Supplement: Supplementary file 1 [file ijms-25-09993-s001.zip › ijms-3169074-supplementary.pdf]

SUPPLEMENTARY MATERIALS

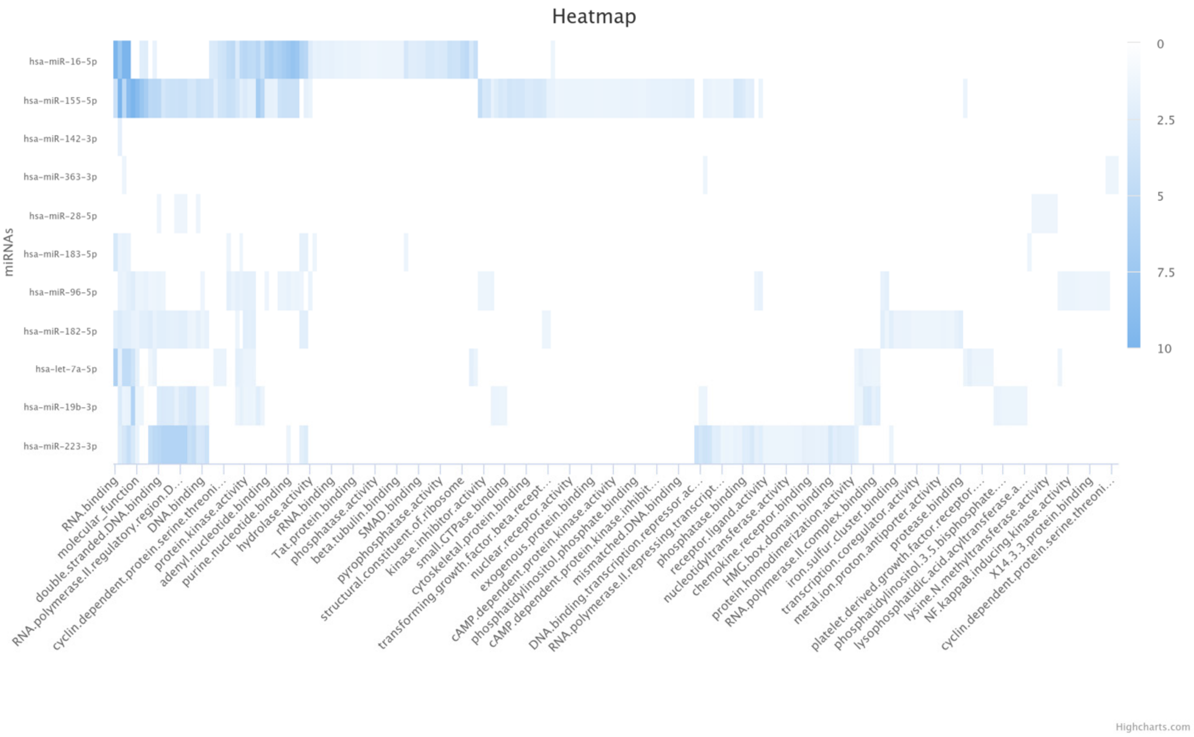

Figure S1. Pathway analysis using gene ontologies based on molecular function using miRPathDB version 2.0 against our 12 miRNA candidates. A higher intensity blue tile denotes a stronger effect on the pathway of the column on the x-axis. The y-axis is composed of the 12 miRNAs identified as markers of Usher from this study.

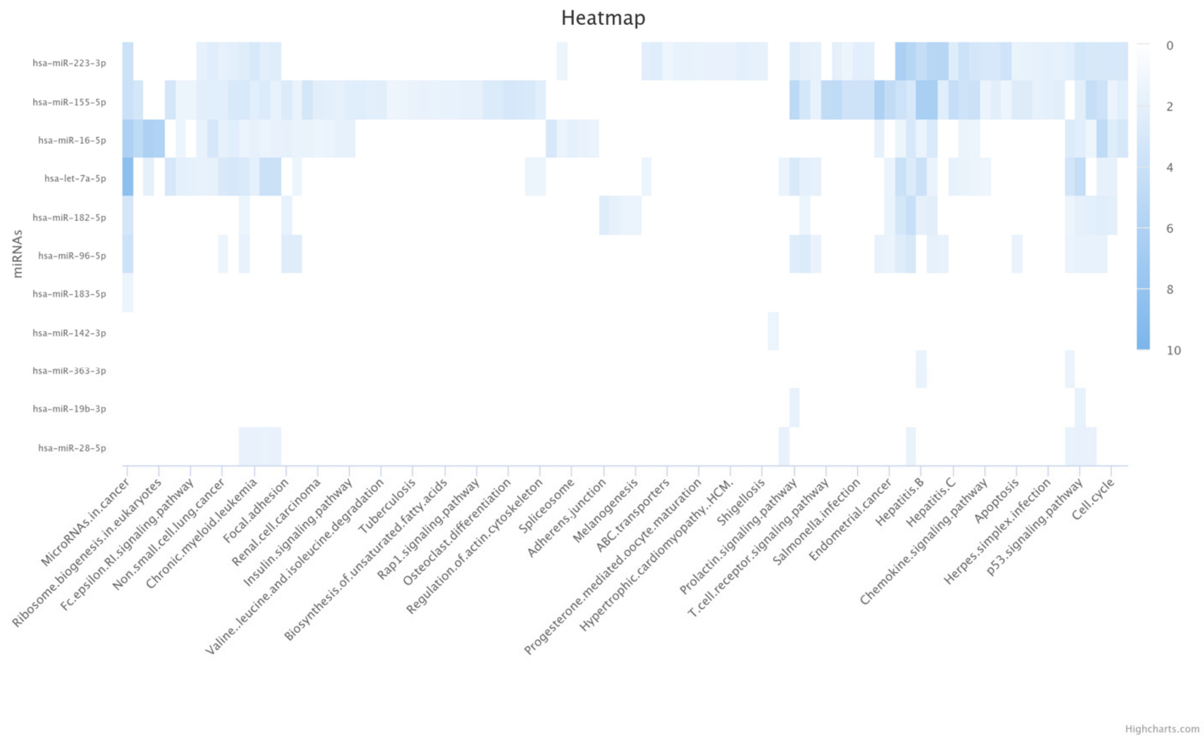

Figure S2. Pathway analysis using KEGG ontologies using miRPathDB version 2.0 against our 12 miRNA candidates. Higher intensity blue tile denotes a stronger effect (see right figure legend) on the pathway of the column on the x-axis. The y-axis is composed of the 12 miRNAs identified as markers of Usher from this study.
